# Temporal trends in refractive error among children and adolescents in Germany – a large-scale analysis of spectacle sales data from dispensing opticians from 2001 to 2025

**DOI:** 10.3389/fpubh.2026.1854832

**Published:** 2026-06-24

**Authors:** Navid Farassat, Sven P. Heinrich, Wolf A. Lagrèze, Niklas Nagel

**Affiliations:** 1 Eye Center, Medical Center, Faculty of Medicine, University of Freiburg, Freiburg, Germany; 2 Department of Economics, University of Freiburg, Freiburg, Germany

**Keywords:** adolescents, children, epidemiology, myopia, refraction

## Abstract

**Background:**

While the prevalence of myopia is rising globally, driven primarily by steep increases in East Asia, recent data on refractive trends in Europe suggest a plateau. This study aimed to evaluate temporal trends in refractive error and myopia progression of children and adolescents in Germany over a 25-year period from January 2001 to December 2025. Specifically, we sought to determine whether there is evidence of an accelerating “myopia epidemic” or a recent pandemic-induced myopic shift.

**Methods:**

This retrospective observational study analyzed a large-scale dataset of real-world spectacle sales sourced from 490 independent dispensing opticians across Germany. It included ~1,250,000 prescription readings from ~440,000 individuals (~49% female) aged 3 to 18 years at baseline. Refractive error was evaluated as spherical equivalent refraction (SER), with myopia defined as ≤ −0.50 diopters (D). Cross-sectional and longitudinal analyses were conducted to evaluate shifts over time in SER, myopic progression, and the influence of age, sex, urbanization, and socioeconomic status.

**Results:**

Contrary to global projections, the overall pediatric spectacle-wearing cohort did not undergo a myopic shift. Instead, a mild hyperopic shift was observed, with the mean SER increasing from +0.05 D in 2001–2005 to +0.53 D in 2021–2025 (*p* < 0.001). Concurrently, the probability of a prescription being myopic decreased by 7.9% over the observation period. Longitudinal analyses of myopic children and adolescents revealed that progression curves remained remarkably parallel, indicating no acceleration in progression rates over the last quarter-century. Peak myopia progression consistently occurred between 8 and 12 years of age. While baseline refraction was a strong predictor for developing high myopia, environmental factors such as urbanicity and socioeconomic status exerted negligible effects on longitudinal progression rates.

**Conclusion:**

This 25-year large-scale analysis found no evidence of an accelerating myopia epidemic or a pandemic-associated surge in myopia progression among spectacle-wearing children and adolescents in Germany. The mean refractive error and the incidence of myopic prescriptions have remained stable or even exhibited a slight hyperopic shift in recent cohorts, suggesting that regional environmental and lifestyle factors may effectively mitigate global myopization trends in this population.

## 1 Introduction

Myopia is increasingly recognized as a major global public health concern with significant socioeconomic consequences, resulting in an estimated global economic burden of approximately 328 billion USD annually (1, 2). The narrative of a global “myopia epidemic” was catalyzed by a widely cited publication by Holden et al. (3), who projected that by 2050, 50% of the global population (4.8 billion people) would be myopic, and 10% (928 million) would be highly myopic. However, these figures were based on extrapolation and did not account for the rising implementation and efficacy of myopia management interventions. Updated models now offer more conservative estimates. For instance, Liang et al. (4) project an overall global prevalence of approximately 40% by 2050, driven by the realization that myopia rates exhibit profound regional variance.

The steepest increases in myopia remain concentrated in East Asia, where prevalence among young adults has surged rapidly from 20–40% before the Second World War to 60–90% today (5–7). In contrast,

Europe has experienced a much slower myopic shift. A recent analysis of the Rotterdam and Generation R cohorts (*n* = 128,012) showed that between 1,900 and 2,000, myopia prevalence increased 2.5 fold from 22% to 56% and high myopia increased 3-fold from 2% to 6% (8). Similarly, an analysis by the European Eye Epidemiology (E3) Consortium, which included over 62,000 European individuals, found an overall myopia prevalence of 31%, rising to nearly 47% among 25- to 29-year-olds (9). Moving forward, prevalence rates in Europe are projected to plateau at around 40% (4).

Within Germany specifically, epidemiological evidence has mostly contradicted the hypothesis of a rapidly accelerating epidemic. The population-based KiGGS study (German Health Interview and Examination Survey for Children and Adolescents), which included survey-based information about the refractive state of over 30,000 children and adolescents, reported that approximately 25% of boys and 35% of girls were myopic by age 17. Crucially, the study found no significant difference in myopia prevalence between the 2003–2006 and 2014–2017 survey periods (10). Corroborating this, Wesemann (11) retrospectively evaluated over 600,000 spectacle prescriptions in Germany and found that the proportion of myopic prescriptions had not increased from 2000 to 2015. In line with this, a recent meta-analysis reviewing myopia prevalence throughout Europe has not found an increase in myopia from 2000 until 2022. Consequently, existing data suggest a stable myopia prevalence among young adults in Germany of roughly 25% to 40%, with insufficient evidence to support a surge over the last two decades.

However, the restrictive lifestyle changes imposed during the COVID-19 pandemic have sparked renewed concerns about “quarantine myopia” (12). Notably, a recent study by Sanz Diez et al. (13) identified a temporary cross-sectional myopic shift among 6- to 11-year-old German children between the pre-pandemic (2015–2019) and pandemic (2020–2021) periods.

To robustly monitor these population-level fluctuations over time, large-scale real-world optician datasets have emerged as highly valuable epidemiological tools (14). The utility of such big data approaches to monitor longitudinal progression has recently been demonstrated by a nationwide cohort study in France (15). In Germany, the large-scale Euronet dataset has proven similarly valuable in the aforementioned studies by Wesemann (11) and Sanz-Diez et al. (13).

Despite these insights, there remains a gap in the literature regarding long-term longitudinal trajectories spanning into the post-pandemic era (2022–2025). The discrepancy between the long-term stability observed by Wesemann and the KiGGS study, and the recent pandemic-associated rise reported by Sanz Diez et al., leaves it unclear whether these fluctuations represent isolated transient perturbations or the beginning of a longitudinal epidemic driven by accelerating digitalization (16).

To address this question, this study leverages 25 years of real-world spectacle sales records (2001–2025) to comprehensively investigate the temporal trends of refractive error in German children and adolescents. Specifically, we aimed to determine whether there has been a recent shift in baseline myopia severity, or an acceleration in myopic progression rates, in the newest birth cohorts compared to those from two decades ago.

## 2 Materials and methods

### 2.1 Study design and data source

This study is a retrospective, observational analysis of anonymized real-world spectacle sales data provided by Euronet Market Research. Euronet offers software solutions specialized for opticians. In exchange for a discount, their customers have the option to assign the rights to their sales data to Euronet. Thereafter, Euronet acts as a data seller, in addition to using the data for their own consulting services. The dataset comprises refractive prescriptions (exclusively of spectacles) and demographic information collected from independent dispensing opticians distributed across Germany. The observation period spans from January 1, 2001, to December 31, 2025. The user base of the software grew from 370 opticians in the initial 2001–2005 period to 490 opticians in the 2021–2025 period. Given the fully anonymized nature of the retrospective data, the Ethics Committee of the University of Freiburg waived the requirement for institutional review board approval in accordance with local regulations. The study adhered to the tenets of the Declaration of Helsinki.

### 2.2 Study population

The analysis included individuals whose age at the time of their initial baseline spectacle purchase was between 3 and 18 years. To ensure data integrity, the following exclusion criteria were applied: Entries were excluded if they contained incomplete refractive data and inconsistent age entries (negative ages, recorded birth before date of visit, age < 3 or > 18 years at baseline). For the analysis of sex and socioeconomic associations, we excluded individuals with missing sex or postal code data, respectively. For longitudinal analyses, only individuals with at least two distinct prescription encounters at minimum 1 month apart were included. Notably, follow-up prescription data for individuals older than 18 were included in longitudinal analyses provided their baseline encounter occurred within the eligible 3- to 18-year age window.

### 2.3 Variables and definitions

Refractive error was calculated as Spherical Equivalent Refraction (SER = Sphere + 0.5^*^Cylinder). Myopia was defined as an SER of ≤ −0.50 D. High myopia was defined as an SER of ≤ −6.00 D.

To analyze historical trends, the dataset was stratified into five 5-year calendar observation periods based on the year of purchase (2001–2005, 2006–2010, 2011–2015, 2016–2020, and 2021–2025).

To examine age-dependent differences, individuals were categorized into four distinct age clusters: 3–6 years, 7–10 years, 11–14 years, and 15–18 years. To investigate geographic and socioeconomic associations, two distinct classification methods were utilized. Urbanization levels (rural, semi-urban, urban) were determined based on the optician's self-reported location category, which was selected by the dispensing optician during their initial software setup. In contrast, the socioeconomic background was estimated at the patient level; the customer's postal code was utilized to categorize socioeconomic status into low, medium, and high strata using the German Index of Socioeconomic Deprivation (GISD).

### 2.4 Statistical analysis

Data processing and statistical analyses were performed using R, packages “car” and “fixest.” Due to the exceptionally large sample size inherent to this real-world dataset, even clinically negligible differences frequently achieved statistical significance (*p* < 0.05). Consequently, results were interpreted with a primary focus on absolute effect sizes and clinical relevance rather than relying solely on *p*-values. Mean SER differences of < 0.25 D were considered clinically negligible. Only data of the right eye of individuals were analyzed. To adjust for potential intra-cluster correlations, which may arise from optician-specific prescribing habits or regional patient demographics, robust standard errors were clustered at the dispensing optician level. Additionally, because customers are nested within opticians, this also accounts for correlation arising from multiple customer visits. To address potential sampling bias introduced by the growing number of opticians using the software over time, a sensitivity analysis was additionally conducted by restricting the dataset to a panel of the original 370 opticians present since the 2001–2005 cohort. An alpha level of 0.05 was established as the threshold for statistical significance.

**Cross-sectional trends:** Changes in mean SER over time were analyzed using Analysis of Covariance (ANCOVA) with clustered standard errors to control for patient age. Point comparisons of mean SER and myopia prevalence at specific educational milestones (ages 6, 12, and 18 corresponding to elementary school entry, transition into secondary education, and secondary school graduation, respectively) across the different calendar cohorts were conducted using Analysis of Variance (ANOVA) and Chi-squared tests with clustered standard errors, respectively. To assess the probability of a prescription being myopic over time, we employed logistic regression models. Because logit estimates can be difficult to interpret clinically, results were converted and reported as Average Marginal Effects (AMEs), expressing differences in percentage points.

**Longitudinal progression analysis:** The temporal progression of myopization was evaluated using linear regression models incorporating a fixed effect per cohort. A continuous time variable (months elapsed since January 2001) was interacted with age groups to assess historical shifts. Wald tests were utilized to evaluate the null hypothesis of equal developmental trajectories (slopes) across different calendar cohorts, sex, and urbanization levels.

**Progression velocity:** The annual progression rate was calculated by dividing the change in SER since baseline by the amount of years passed since baseline. The relationship between age at first prescription, historical time period, and the annual rate of myopia progression was modeled using polynomial regression (incorporating a squared term for baseline age, age^2^). This allowed for the identification of the “peak” age of myopic progression. Historical comparisons of maximal progression velocity were conducted using ANOVA with clustered standard errors.

**Risk modeling for high myopia:** A logistic regression model was utilized to calculate the 10-year probability of progressing to high myopia. Baseline SER (grouped into severity bins) and baseline age (coded as dummy variables to capture non-linear effects) were set as independent predictors. Outcomes for this model were similarly reported using relative AMEs to convey the risk difference in percent.

## 3 Results

### 3.1 Demographics

A total of 1,254,678 prescriptions from 437,711 individuals (49.36% female) were analyzed. 1,167,815 prescription readings were included in the cross-sectional analyses and 461,920 readings in the longitudinal analyses (average time interval between longitudinal visits = 1.4 years). The mean age at baseline was 11.3 years while the mean age of all prescriptions readings was 14.1 years. Furthermore, the mean age at the time of the first prescription decreased steadily across the observation periods: 12.0 years in 2001–2005, 11.3 years in 2006–2010, 11.2 years in 2011–2015, 10.9 years in 2016–2020, and 10.7 years in 2021–2025.

### 3.2 Overall temporal trends in refractive error (all children and adolescents)

**Figure 1:**
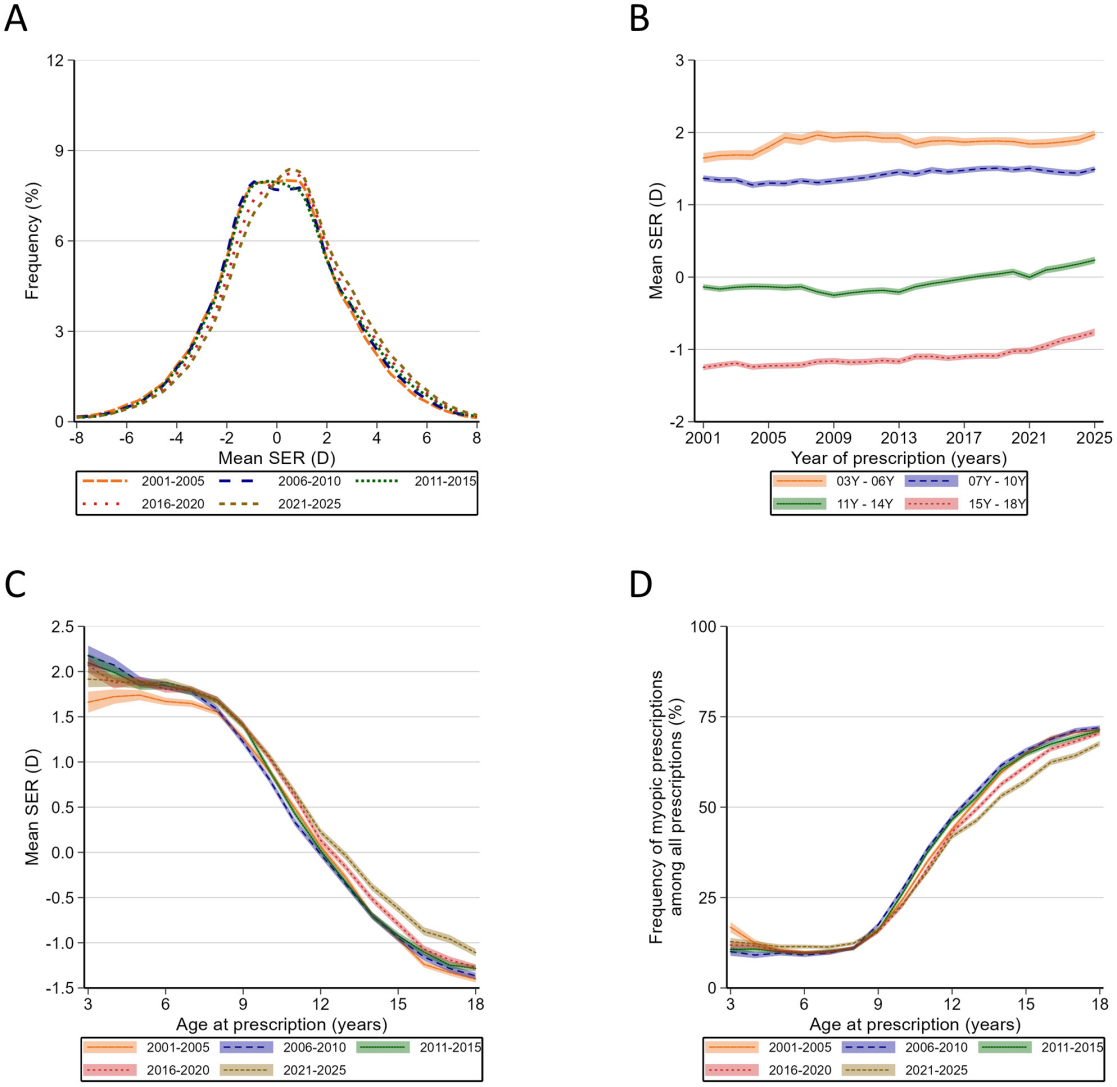
Refractive error distribution and cross-sectional trends across different time periods. **(A)** Relative distribution of Spherical Equivalent Refraction (SER) across the five observation periods. **(B)** Mean SER over the 25-year observation period, stratified by age groups. **(C)** Cross-sectional analysis of mean SER by age at prescription. **(D)** Proportion of myopic prescriptions among spectacle wearers as a function of age. Shaded areas in **(B)**, **(C)** and **(D)** represent 95% confidence intervals.

We performed a cross-sectional analysis of all prescriptions (including not only myopic but also hyperopic and astigmatic prescriptions) for children and adolescents across the 25-year observation period which revealed no trend toward increasingly myopic spectacles in the overall pediatric spectacle-wearing population aged 3 to 18 years. In fact, we observed a statistically significant, though clinically mild, hyperopic shift. The mean SER of all analyzed prescriptions increased from +0.05 D in the 2001–2005 period to +0.53 D in 2021–2025 (*p* < 0.001) (Figure 1A).

When controlling for age, this shift remained consistent (Figures 1B, C). In groups stratified by age, mean SER increased slightly in all age groups except in the youngest children aged 3–6 years (Figure 1B, 3–6 year old children: *p* = 0.055, all other age groups: *p* < 0.001). Point-comparisons at key educational milestones further illustrate this stability: from 2001 to 2025, the mean SER at school entry (age 6) shifted slightly from +1.67 D to +1.88 D (*p* < 0.001); at middle school age (age 12) from +0.07 D to +0.22 D (*p* < 0.001); and at school-leaving age (age 18) from −1.40 D to −1.11 D (*p* < 0.001) (Figure 1C).

Correspondingly, the probability of receiving a myopic prescription slightly decreased in the most recent cohorts (Figure 1 D). At average marginal effects, the probability of a prescribed spectacle being myopic was 7.87 percentage points lower in 2021–2025 compared to 2001–2005 (*p* < 0.001) (Figure 1D). For example, the share of myopic prescriptions among 18-year-olds dropped from 71.40% in 2001–2005 to 67.62% in 2021–2025 (*p* < 0.001).

### 3.3 Temporal dynamics and progression rates in myopic children and adolescents

**Figure 2:**
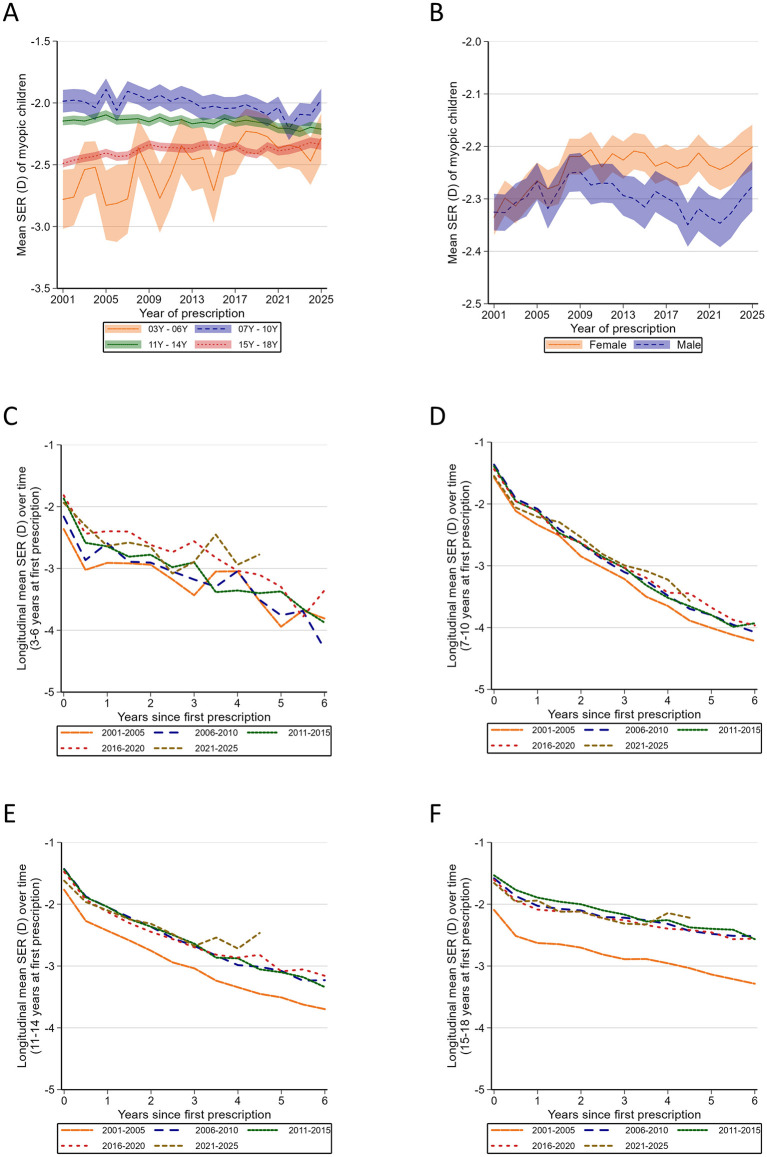
Cross-sectional refractive trends and longitudinal SER trends among myopic children and adolescents. **(A)** Mean Spherical Equivalent Refraction (SER) of myopic individuals over time, stratified by age groups. **(B)** Mean SER of all myopic individuals (aged 3–18 years) by year of prescription stratified by sex. **(C–F)** Longitudinal myopic trends, depicted as mean SER against the number of years since the first spectacle prescription, stratified by 5-year calendar cohorts. Age groups are defined by age at first prescription: **(C)** 3–6 years, **(D)** 7–10 years, **(E)** 11–14 years, and **(F)** 15–18 years. Shaded areas represent 95% confidence intervals.

When restricting the analysis strictly to individuals with established myopia (SER ≤ −0.50 D), temporal trends showed similarly stable or even slightly less myopic SERs in 2025 compared to 2001 for all distinct age groups (3–6 years: from −2.78 D in 2001 to −2.28 D in 2025, *p* < 0.001; 7–10 years: from −1.99 D in 2001 to −1.97 D in 2025, *p* = 0.0983; 11–14 years: from −2.15 D in 2001 to −2.21 D in 2025, *p* < 0.05; 15–18 years: from −2.49 D in 2001 to −2.34 D in 2025, *p* < 0.001; Figure 2A). A sex–stratified analysis revealed that males exhibited a lower (more myopic) SER than females overall (*p* < 0.01); however, the difference was clinically negligible (mean difference = 0.05 D, Figure 2B). Only for females, we observed a slightly less myopic mean SER in 2025 compared to 2001 (2001: −2.33 D, 2025: −2.20 D, *p* < 0.05) leading to a maximum difference of 0.11 D between sexes (in 2019 and 2020).

To determine if myopia is progressing faster today than two decades ago, we analyzed longitudinal trajectories by age group (Figures 2C–F). While there were some historical differences in the “start point” of myopia (e.g., older adolescents in the 11–14 and 15–18-year age brackets exhibited notably higher baseline myopia in the initial 2001–2005 cohort compared to other cohorts, SER 0.40 D more myopic in 2001–2005 compared to 2021–2025, *p* < 0.001), the slopes of the longitudinal trajectories remained remarkably parallel across all historical cohorts (in D/year; 3–6 years: 2001–2005: −0.23, 2006–2010: −0.29, 2011–2015: −0.31, 2016–2020: −0.28, 2021–2025: −0.31; 7–10 years: 2001–2005: −0.47, 2006–2010: −0.48, 2011–2015: −0.47, 2016–2020: −0.45, 2021–2025: −0.47; 11–14 years: 2001–2005: −0.35, 2006–2010: −0.33, 2011–2015: −0.33, 2016–2020: −0.32, 2021–2025: −0.32; 15–18 years: 2001–2005: −0.21, 2006–2010: −0.18, 2011–2015: −0.18, 2016–2020: −0.18, 2021–2025: −0.21). Across all age groups, children aged 7–10 years exhibited the steepest myopization slopes. Although Wald tests evaluating trajectory interactions reached statistical significance in the older age groups (11–14 years and 15–18 years, *p* < 0.001), this is primarily due to the immense sample sizes (3–6 years: *n* = 24,532, 7–10 years: *n* = 72,995, 11–14 years: *n* = 186,648, 15–18 years: *n* = 177,745).

### 3.4 Longitudinal progression and risk factors for high myopia

**Figure 3:**
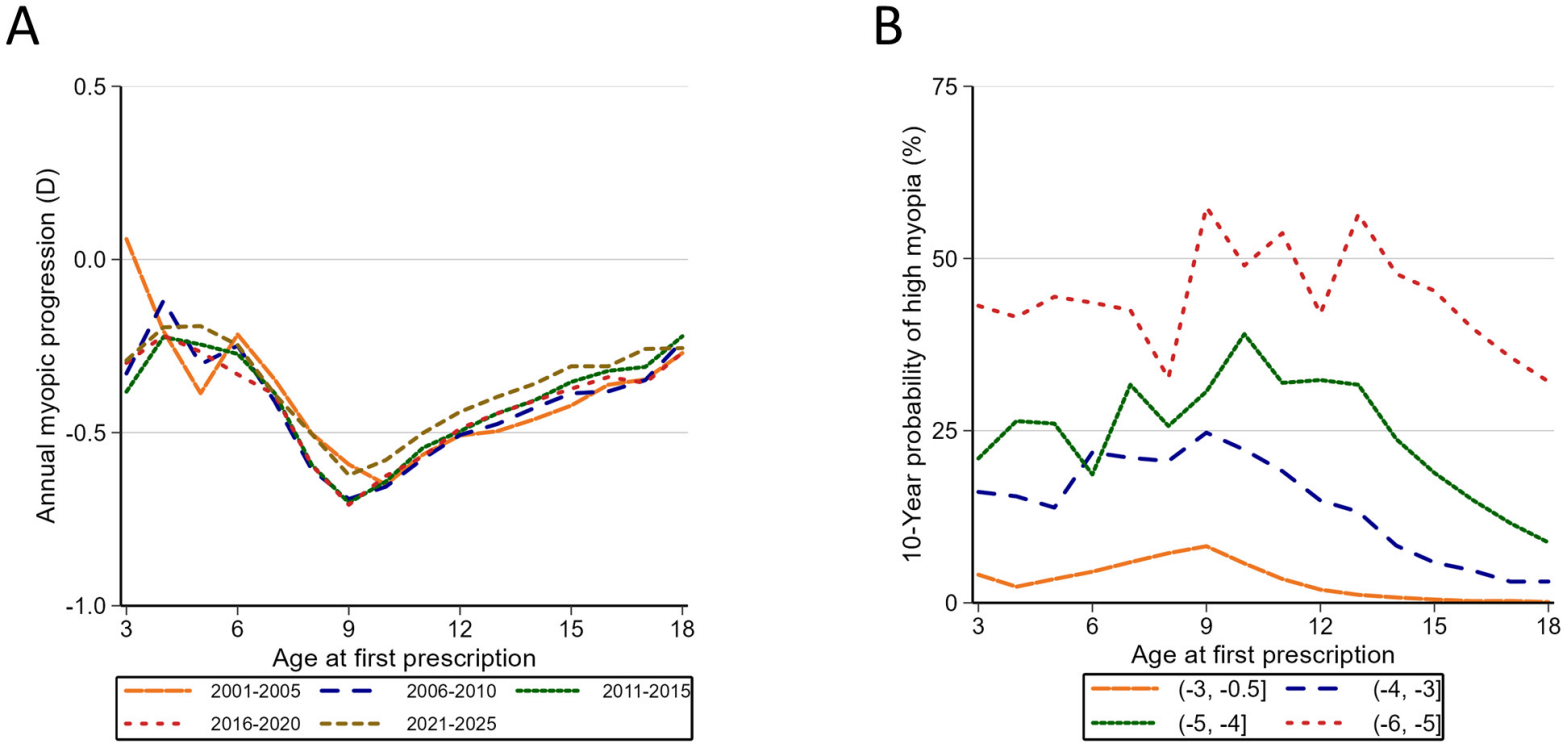
Annual progression rates and the probability of developing high myopia. **(A)** Average yearly change in Spherical Equivalent Refraction (SER) among myopic individuals as a function of age at first prescription, stratified by 5-year historical cohorts. **(B)** 10-year probability of developing high myopia (SER ≤ −6.00 D) based on age at first prescription, stratified by the initial severity of myopia.

Annual progression rates exhibited similar age-dependent maxima occurring at around 8–12 years of age (Figure 3A). Peak progression age shifted slightly from 11.12 years in 2001–2005 to 10.15 years in 2021–2025, while absolute maximum progression rates remained relatively stable (e.g., −0.57 D/year in 2001–2005 vs. −0.55 D/year in 2021–2025 for 7–10–year–olds, *p* < 0.001; −0.38 D/year in 2001–2005 vs. −0.30 D/year in 2021–2025 for 15–18–year–olds, *p* < 0.001).

A logistic regression analysis was conducted to evaluate predictors for developing high myopia (SER ≤ −6.00 D) over a 10–year period (Figure 3B). Baseline refraction proved to be a superior predictor compared to the age of onset. At average marginal effects and after adjusting for age at first prescription, individuals with a starting refraction of −5.00 to −6.00 D had a 1389% higher risk of developing high myopia compared to those starting between −0.50 and −3.00 D (*p* < 0.001). In contrast, age at first prescription was a weaker predictor; for example, presenting at the peak progression age of 10 years increased the probability of high myopia by 72.46% compared to presenting at age 6 (*p* < 0.001) after adjusting for baseline refraction. This is probably due to a limitation inherent to our study design: Within our retrospective dataset, the observational follow-up times were much shorter for younger children presenting at the optician. Specifically, the average follow-up was 3.5 years for the baseline age group of 6–9 years, compared to 4.5 years for the 9–12 years age group.

### 3.5 Environmental and socioeconomic associations

**Figure 4:**
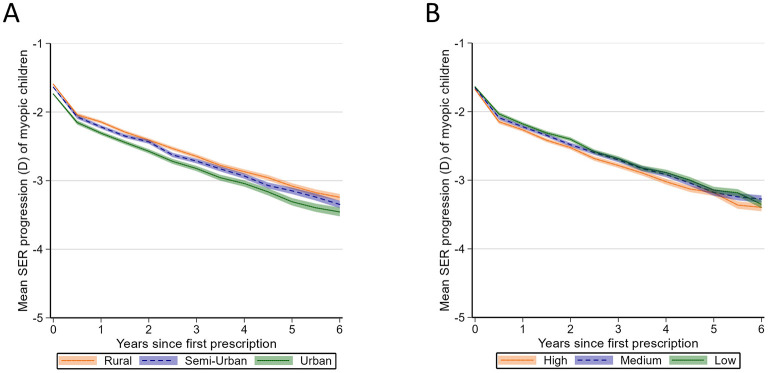
Influence of urbanization and socioeconomic status on myopia progression. Longitudinal progression of mean Spherical Equivalent Refraction (SER) from the time of first prescription over a 6-year follow-up period, stratified by **(A)** urbanization level (in lower case: rural, semi-urban, urban) and **(B)** socioeconomic status (low, medium, high). Shaded areas represent 95% confidence intervals.

We identified a weak relationship between urbanization and initial myopia severity (Figure 4A). Children and adolescents residing in urban areas presented with a small but significantly more myopic baseline SER compared to their rural peers (*p* < 0.001). However, the longitudinal progression curves for urban, semi-urban, and rural environments ran virtually parallel (in D/year; rural: −0.29, semi–urban: −0.30, urban: −0.31), though – again due to the immense sample size – we found statistical significance in the Wald test evaluating trajectory interactions (*p* < 0.05). This indicates that the urban environment mostly affects the onset or initial magnitude of refractive error and less so the speed of progression once myopia is established. Socioeconomic status (SES) had minimal, though statistically significant impact on baseline SER (*p* < 0.01 for low vs. high SES) but no effect on progression rates (in D/year; low: −0.30, medium: −0.30, high: −0.31) (Figure 4B).

**Sensitivity Analysis** Across all cross-sectional, longitudinal, and environmental analyses evaluated in this study, the observed outcomes were highly robust to potential shifts in the sample composition over the 25-year period. Notably, these temporal trends remained stable even when limiting the dataset in a sensitivity analysis to the panel of 370 opticians present since the first observation period (data not shown).

## 4 Discussion

The primary objective of this large-scale retrospective study was to evaluate the temporal trends in refractive error among children and adolescents in Germany over a 25-year period. Contrary to the widespread narrative of a rapidly accelerating global myopia epidemic (3), we found no increase in myopic prescriptions between 2001 and 2025. Instead, a statistically significant, albeit clinically mild, trend toward hyperopization was observed in the most recent observation period (2021–2025).

These results closely align with shorter-term epidemiological data from Germany, such as the population-based KiGGS study, which demonstrated a stable myopia prevalence among German children and adolescents between the 2003–2006 and 2014–2017 survey waves (10). Similarly, our data substantiate the findings of Wesemann (11), who reported no increase in the proportion of myopic spectacle prescriptions in Germany from 2000 to 2015. Crucially, our analysis extends this stable trajectory up to the year 2025. While Sanz Diez et al. (13) recently utilized a subset of the Euronet dataset to identify a transient cross-sectional myopic shift among 6- to 11-year-olds during the immediate COVID-19 confinement period (13), our 25-year longitudinal analysis demonstrates that this did not translate into a sustained acceleration of myopia progression. This argues against concerns that the COVID-19 pandemic and accelerating digitalization have triggered a quarantine myopia surge in Germany as reported in other studies primarily from Asia (12, 17). Ultimately, our findings support a recent European meta-analysis (18) indicating that myopia prevalence in Europe has largely plateaued since 2000. The severe environmental pressures driving the East Asian myopia boom, such as extreme and early educational intensity and a severe lack of outdoor time (19, 20), may be less pronounced, or more effectively counterbalanced, within the German system and lifestyle (21, 22).

Beyond overarching temporal trends, our analysis revealed several findings regarding myopia progression and demographics. We observed a minor, clinically negligible sex difference (0.05 D), with males presenting with slightly more myopic refractions. While this opposes recent international studies which frequently report female sex as a risk factor for higher myopia prevalence and faster progression rates, it aligns with older epidemiological research suggesting these differences reflect evolving socio-environmental influences, such as changing access to education and differential engagement in near-work or outdoor activities (23).

Accordingly, in Orthodox Jewish communities in Israel, where boys undergo intensive religious education, they have a significantly higher prevalence of myopia than girls (24). Regarding longitudinal dynamics, our data show that the maximum myopia progression occurs between the ages of 8 and 12 years. This is consistent with the findings of Tricard et al. (15), who, in a nationwide longitudinal prospective study in France, also identified children aged 10 to 12 years as exhibiting the most rapid progression rates. While physiological eye growth is most rapid earlier in life [ages 6–9; see Tideman et al. (25)], axial elongation accelerates significantly and is fastest in the three years just prior to myopia onset (26). Therefore, peak refractive myopia progression (in D) may occur later than peak axial elongation.

When modeling the 10-year probability of developing high myopia, baseline refraction emerged as the dominant predictor, while the age at first prescription appeared less impactful. However, early onset remains a critical recognized risk factor in the broader literature (23), and its impact here may be underestimated due to shorter observational follow-up times for the youngest cohorts in our dataset.

Furthermore, in our cohort, urbanicity and SES exerted negligible longitudinal effects on progression rates, even though urbanization was associated with slightly higher initial myopia severity. This distinction aligns with evidence suggesting that environmental factors primarily influence disease onset rather than subsequent progression velocity (27, 28). However, the overall lack of an urban-rural or SES disparities in our data contrasts with several global studies. In other populations, urban environment and higher SES are strongly linked to increased myopia risk, typically driven by associated environmental pressures such as intensive education and near work (29), increased screen time (30), and limited outdoor time (23). The negligible difference between urban and rural myopia progression in our cohort may be attributed to Germany's highly decentralized and largely homogenous infrastructure as well as its relatively standardized educational system, which mitigate the extreme urban-rural lifestyle disparities often observed in East Asian cohorts. Furthermore, urbanization was subjectively self-categorized by opticians based on store location rather than customer residence. Since rural customers may travel to urban opticians (and vice versa), this geographic proxy introduces a potential misclassification bias.

Our findings must be considered in light of several methodological limitations. First, the retrospective nature of the dataset relies on real-world dispensing optician records rather than standardized clinical examinations. Therefore, we assume that the refraction data were at least partially non-cycloplegic. Because pediatric eyes have a strong accommodative capacity, non-cycloplegic autorefraction and subjective refraction frequently overestimate myopia rendering the measured values too myopic (31). This is especially true for the youngest children in this cohort (3–6 years) and may partially explain the relatively high myopia in this age group. However, assuming this accommodative bias has remained relatively constant over the 25-year observation period, the longitudinal and temporal trends we report remain valid. Second, the dataset lacks accompanying clinical information regarding general ocular health, biometry (axial length), and parental myopia. Axial length is known to be the more robust parameter for monitoring myopia progression for several reasons: modern optical interferometry offers significantly higher sensitivity, lower test-retest variability, and fewer diurnal fluctuations compared to refraction measurements (32). Furthermore, axial elongation provides a direct structural measure of eye growth that is unconfounded by compensatory changes in the anterior segment, and it represents the primary risk factor for myopia-associated secondary complications and visual impairment (33). Third, there is an inherent selection bias: our data only include children and adolescents who visited an optician and received a refractive correction, meaning emmetropic children are entirely excluded from this analysis. It remains elusive whether hyperopes or myopes are disproportionately underrepresented overall. For example, myopes might be underrepresented if young children fail to recognize or articulate poor distance vision. On the other hand, it is also plausible that myopes are actually overrepresented in the youngest age group (3–6 years) in our cohort. This may be due to a combination of factors: (1) the higher accommodative amplitude in younger children leading to non-cycloplegic overestimation of myopia, as described above; (2) statistical artifacts as a consequence of the smaller sample size in this very young cohort; and (3) the likelihood that cases of genetic or pathological early-onset myopia are overrepresented in this age group, as typical “school myopia” usually develops later. These factors collectively contribute to the aforementioned relatively high myopia observed in this age group. In addition, the slight hyperopic shift observed in the most recent cohort (2021–2025) compared to earlier cohorts (2001–2005) might not reflect a true biological population shift. Instead, it may reflect a changing threshold for prescribing spectacles. Parents and practitioners may be more readily correcting mild hyperopia or astigmatism today due to heightened awareness of pediatric refractive errors and more proactive screening over time. In line with this, we found that the mean age at first prescription steadily decreased from 12.0 years in the earliest cohort (2001–2005) to 10.7 years in the most recent cohort (2021–2025). Fourth, an artifact related to the database being initialized in 2001 likely explains the distinctly higher baseline myopia levels observed in the older age groups (Figures 2E, F) between 2001 and 2005. At the inception of the dataset, an older adolescent visiting an optician was recorded as a “new” baseline entry. This inadvertently mixed true late-onset myopes with earlier-onset myopes who had already progressed to higher levels of myopia for years prior to their first recorded visit. In later historical cohorts (e.g., 2016–2025), teenagers who developed myopia early in childhood would have likely been captured by the database at a much younger age. Consequently, the 15–18-year age bracket in more recent years consists almost exclusively of true late-onset myopes, who naturally present with much milder initial myopia. Fifth, our dataset is derived from 490 independent dispensing opticians, which represents approximately 5% of the German market. This sample inherently excludes large corporate retail chains that account for a significant portion of spectacle sales in Germany. While we have no reason to assume that the underlying biological refractive errors of children visiting independent opticians differ fundamentally from those visiting retail chains, differences in socioeconomic status might exist, as corporate chains often compete heavily on price. Nevertheless, as demonstrated in our analysis (Figure 4B), the impact of socioeconomic status on baseline refraction and progression rates was found to be negligible, suggesting that this specific sampling limitation is unlikely to meaningfully alter our overarching conclusions. Finally, the study employs a mixture of cross-sectional and longitudinal analyses, which requires careful interpretation when extrapolating individual progression risks from population-level cross-sectional means.

In conclusion, this large-scale analysis of spectacle sales data provides reassuring evidence regarding the refractive health of children and adolescents in Germany. Over the past 25 years, we found no indication of an accelerating “myopia epidemic” or an increase of myopia progression rates. The mean refractive error and the probability of receiving a myopic prescription have remained stable, with recent cohorts even exhibiting a slight hyperopic shift. While clinical variables such as baseline refraction strongly dictate the risk of future high myopia, broad environmental factors like urbanicity and socioeconomic status exerted negligible longitudinal effects in this cohort.

## Data Availability

The data analyzed in this study is subject to the following licenses/restrictions: The data analyzed in this study were obtained from a third-party commercial provider (Euronet Market Research, part of the EVEX group) under a strict licensing agreement. Due to contractual obligations and legal restrictions prohibiting the distribution or sharing of this purchased dataset, the raw data cannot be made publicly available. Researchers who wish to access the underlying data must obtain it directly from the commercial provider. Details regarding the analytical methods and protocols used to conduct this research are described in the Materials and Methods section, and further information regarding the statistical analysis can be made available by the corresponding author upon reasonable request. Requests to access these datasets should be directed to https://www.evex-group.com/.
